# Remarks on Article on Foul Breath

**Published:** 1879-05

**Authors:** 


The paper republished from the *Missouri Dental Journal* in the last number of this Journal, on "Foul Breath," is of exceeding interest, and the more so because of the commonly avoided discussion of the topic. The dentist should aim to come to his patients with as little that is disagreeable about him as is possible. He is a badly tolerated infliction at best, but when standing before his patient with breath reeking with foul odors, he is simply disgusting. To him, however, who from unavoidable causes, as dyspepsia, &c., is burdened with a trouble of this sort which all his care will not eradicate, a few hints may be useful.

The seat of most foul breath, of constitutional origin, consumptives excepted, *is located at the base of the tongue.* Open the mouth, protrude the tongue, and note, in cases of offensive breath, the coating at the root of that organ : that coating is mainly the lodging place of the trouble. A stiff tooth brush, a little fine castile soap, and scrubbing, will bring about cleanliness the sufferer little hoped for. It is quite astonishing what great relief this simple act of cleaning will bring. If further help is required, a grain or two of crystallized carbolic acid in a glass of water as a gargle, will almost always remedy the evil for the time, for of course this relief is but temporary, and can only be eradicated by the removal of the primary cause.

Constipation not unfrequently causes foul breath. The sufferer is often not aware of this as a cause, yet it is one. The remedy of course is not purgatives, but *fruits and grains.*

A *candid mentor* is most useful in such cases. It is not in good taste to consult one's acqaintance on the subject. An evasive answer is almost invariably given ; and then too the sufferer from this malady is, as a rule, unaware of its existence. Consult your wife, sister, mother, and do this frequently, not waiting for them to call your attention to the trouble, but invite criticism. The olfactories of women are more sensitive than those of men.

Chronic Catarrhal troubles and the resulting ozæna, are of so grave a nature as to call up the question whether he who has disease of so serious tendencies should practice a profession such as dentistry—a calling making heavy demands on the vitality. Open air occupation, persistently, patiently followed, should be the remedy in the main. It is a mistake that dentistry is a profession which delicate men may follow without harm ; and a serious error that because one is consciously too feeble to follow an active out-of-doors occupation, that therefore dentistry may be pursued as easier. Many a life is thus lost. H.

